# Malformation of the Uterus: One Kidney Deficient: Ptyalism Caused by the Internal Use of Bismuth

**Published:** 1846-04

**Authors:** John Cardew

**Affiliations:** Physician to the Bath Hospital


*Malformation of the Uterus: one Kidney Deficient: Ptyalism caused by the Internal use of Bismuth.* By John Cardew, M. D., Physician to the Bath Hospital.—Ann Hitchings, aged 38, was received into the hospital July 7, 1845, upon the recommendation of Mr. Thomas Warner, of Cirencester, by whom, conjointly with Mr. Charles Pooley, the following interesting history of her case was kindly furnished.

She was four feet two inches in height, and always of an active bustling disposition, up to the commencement of her first illness, eight years ago. She did not menstruate till between the twentieth and twenty-first year, and afterwards very irregularly, the interval between each return varying from a week to five months. The catamenia were always scanty and pale, but unattended with pain or uneasiness of any kind. The statements with regard to the urine were conflicting; one account representing it to be copious, very pale and inodorous, and slightly albuminous; while the patient's mother-in-law now asserts that it was always scanty, and that she had frequently known her pass a whole day without voiding any. During the time she was in the hospital, the secretion, both as to quantity and quality, agreed with the former of these representations. She attributed the origin of her complaint to a violent cold caught by gleaning in a wet field; when she began to suffer from a dull aching in the loins, disabling her from lifting anything weighty, and accompanied by a sharp lancinating pain, which she described as shooting all over her, and, for a minute, taking away the use of her legs. These shocks recurred as often as four or five times during the day, and unless she was near any support, she generally fell. Her arms were sometimes affected, but not often. Her principal uneasiness was confined to the loins, and not at all to the spine itself. About two years after her marriage she became very large, and from the suppression of the catamenia during seven months, thought that she was pregnant, but the swelling gradually subsided. Since then she had only menstruated two or three times, and five years had elapsed since the last appearance of that discharge. Her husband stated that she was quite like other women in regard to sexual desires. Her countenance was pale and sallow; she complained greatly on being touched, and screamed aloud if the fingers were passed rapidly over the abdomen; but firm pressure caused her no uneasiness. For some time she had been subject to twitchings of the muscles of the face, and frequent winking of the eyes, with intolerance of light; but her head had never been much affected. She had also, at different times, attacks of what might be called congestive pneumonia, but, on two or three occasions, they partook apparently of a more active character, attended wiih membranous inflammation, so that venesection was had recourse to, when the blood presented the usual buffy aspect, and once or twice to a great degree.

At the time of her admission into the hospital, her skin was dry, and rather hot; her tongue clean; bowels rather costive; pulse averaging 84, small and easily compressible; appetite good; auscultation good throughout. The singular screwing up of the features and winking of the eyelids, seemed to proceed from some affection of the base of the brain; while the impaired motor powers, and excessive sensibility of the integuments of the abdomen, appeared to have their origin in a morbid condition of the spinal membranes; but as she had neither pain nor heat in the head, nor tenderness at any part of the spine, and as at an earlier period she had been subjected to local depletion, and counter-irritation, it was considered that no active disease was in progress, and that the treatment should be directed to repairing the consequences of former inflammatory action. With this impression she was directed to use the bath, and her bowels were regulated by aloetic aperients; and after a time, she was allowed to drink the Bath waters.

Up to the 21st of August there was little change in her state, but as she then complained much of gastrodynia, she was ordered to take, twice a day, five grains of trisnitrate of bismuth, with a cordial alkaline draught. This treatment was continued until the 26th, when a profuse salivation supervened, precisely resembling, in the appearance of the mouth and fœtor, that occasioned by mercury. She was now kept in bed, had her bowels attended to, used gargles, and had nourishing slops, with eggs, &c.; to which was added, on the 4th of September, four ounces of sherry daily. On the 12th and 13th her mouth began to mend, and her general state to become more comfortable; but on the 14th, her evacuations having passed involuntarily, it was necessary to change her linen and bed-clothes. As usual when moved, she complained bitterly of this process, but did not apparently suffer from it until the evening. Mr. George was called to her at nine o'clock, when her extremities were cold, her pulse fluttering, and her breath had that stertorous character which is met with in scarlatinous patients, whose throats are badly effected. Sinapisms, hot bottles to the extremities, and internal stimulants failed in exciting any reaction, and she died at ten the next morning.

*Examination thirty hours after death.*—The brain and the membranes investing its upper surface were perfectly healthy; but the pia mater at the base, showed much vascularity, and the vessels became more numerous where that membrane was continued over the medulla oblongata; in the right lateral ventricle were about ten drachms of serum, and twenty in the left; the pia mater was much injected throughout the whole length of the spinal cord, the vascularity being most intense at the commencement of the cauda equina; the theca vertebralis contained six drachms of fluid. There was no effusion into the cavity of the chest; the lungs exhibited some venous engorgement, but were not otherwise unhealthy; the heart flabby, and, like the other viscera, small. Notwithstanding the general emaciation of the limbs, there was a thick layer of fat upon the muscles of the abdomen, as well as in the omentum and mesentery; the stomach and intestines were healthy; the left kidney was small and pale, but not granular. On the right side *no vestige of a kidney could be traced.*

The uterus was divided into two portions, connected by a narrow neck; the lower part, in the centre of which was the orifice, was of about the size of a cobnut; the upper part, an inch and a half in length, was of the thickness of the little finger of a small hand.—*Prov. Med and. Surg. Journ.*

